# First record of pigmentation disorder in the Fringe-lipped Bat *Trachops cirrhosus* (Spix, 1823) (Chiroptera: Phyllostomidae) from southeast Brazil

**DOI:** 10.3897/BDJ.7.e38304

**Published:** 2019-08-28

**Authors:** Ianna Sonegheti Borloti, Vinícius Teixeira Pimenta, Albert David Ditchfield

**Affiliations:** 1 Centro de Investigação em Biodiversidade e Recursos Genéticos da Universidade do Porto (CIBIO-UP). Departamento de Biologia, Faculdade de Ciências da Universidade do Porto, Porto, Portugal Centro de Investigação em Biodiversidade e Recursos Genéticos da Universidade do Porto (CIBIO-UP). Departamento de Biologia, Faculdade de Ciências da Universidade do Porto Porto Portugal; 2 Centro de Ciências Humanas e Naturais. Departamento de Ciências Biológicas, Universidade Federal do Espírito Santo - UFES, Vitória, Brazil Centro de Ciências Humanas e Naturais. Departamento de Ciências Biológicas, Universidade Federal do Espírito Santo - UFES Vitória Brazil

**Keywords:** Aberrant coloration, abnormal coloration, anomalous color, Atlantic Forest, chromatic disorder, piebaldism, Phyllostominae.

## Abstract

Piebaldism is a genetic pigmentation disorder, which is caused by absence of melanocytes in parts of the skin and/or hair follicles, with eyes and claws normally pigmented. The occurrence of piebaldism in natural populations is rare and the effects on fitness are still unknown. This article reports the first case of pigmentation disorders in the Fringe-lipped Bat *Trachops cirrhosus* (Spix, 1823) (Chiroptera: Phyllostomidae) caught in Barra do Triunfo, city of João Neiva, northeastern state of Espírito Santo, southeast Brazil.

## Introduction

Records of the occurrence of pigmentation disorders in natural populations has grown worldwide (Uieda 2000; Zalapa et al. 2016). Piebaldism is the condition in which the absence of melanin is localized, caused by the absence of melanocytes as a result of genetic mutations, and affects skin and hair follicles. Piebald individuals have a variable distribution of white patches on the body, but the eyes are normally pigmented (Abreu et al. 2013). According to a review of pigmentation anomalies in bats proposed by Lucati and Lópes-Baucells (2016), this condition is normally misclassified as leucism, which is the complete absence of pigmentation in the entire body except on the eyes, which are always normally coloured.

The adaptive significance of coloration in mammals is associated with concealment from potential prey or predators, communication and regulation of physiological processes (Caro 2005). There is a lack of knowledge about evolutionary mechanisms responsible for these aberrant phenotypes and their possible costs and benefits (Bilandžija et al. 2013). Individuals that have pigmentation disorders are expected to show reduced fitness, because the phenotype appears at low frequency in natural populations. In fact, some authors consider that chromatic disorders lead to overexposure and higher predation risk (Marín‐Vásquez et al. 2010). Other authors, however, argue that, in dark environments, selection for pigmentation is relaxed, resulting in a variety of colorless forms. Because bats generally select dark roosts and forage at night, their coloration may have no effect on predation or social behaviour (Buys et al. 2002).

Chromatic disorders in bats have been reported worldwide in at least 609 individuals belonging to 115 species and 10 families (Lucati and López-Baucells 2016). In Brazil, cases of piebaldism have been reported only for the families Molossidae and Phyllostomidae, in the species *Nyctinomops laticaudatus* (É. Geoffroy, 1805), Tadarida brasiliensis (I. Geoffroy, 1824), *Artibeus concolor* Peters, (1865), *Artibeus jamaicensis* Leach, 1821, *Artibeus lituratus* (Olfers, 1818), *Carollia perspicillata* (Linnaeus, 1758), *Phyllostomus discolor* Wagner (1843) and *Tonatia saurophila* Koopman & Williams (1951) (Geiger and Pacheco 2006; Rocha et al. 2013; Souza et al. 2013; Treitler et al. 2013; Guimarães et al. 2014; Lucati and López-Baucells 2016). Chromatic disorders had never been reported in the species *Trachops cirrhosus* (Uieda 2000; Abreu et al. 2013; Lucati and López-Baucells 2016). This record increases to nine the number of bat species reported with piebaldism in the Brazilian territory.

## Methods

The capture of bats was performed using mist nets placed at ground level inside a small cave in Barra do Triunfo, city of João Neiva, northeastern state of Espírito Santo, southeast Brazil (19°41'35"S and 40°22'17"W, elevation of 204 m), in May 2010. The climate is classified as Tropical (Am in Koppën's classification), with a rainy season in the summer and a short dry season in the winter. Part of the Atlantic Forest Biome, the dominant vegetation is tropical rainforest. The native vegetation of the region is highly fragmented and the cave is located at the edge of a forest fragment.

Two mist nets were opened before nightfall, to intercept the departure of bats from the shelter. Two individuals of *T. cirrhosus* were collected as voucher specimens and taken to the "Laboratório de Estudos em Quirópteros" (LABEQ) of Universidade Federal do Espírito Santo (UFES), where they were killed following the cervical dislocation protocol, fixed in 10% formalin and preserved in 70% ethanol in the mammal collection.

## Results

Four species were identified, three in the family Phyllostomidae: *Anoura geoffroyi* Gray, 1838 (n = 13), *Desmodus rotundus* (É. Geoffroy, 1810) (n = 1), *Trachops cirrhosus* (n = 2) and one in Vespertilionidae: *Myotis levis* (I. Geoffroy, 1824) (n = 1). In this cave, a chromatic disorder was recorded only in one specimen of *T. cirrhosus*.

**Figure 1.**
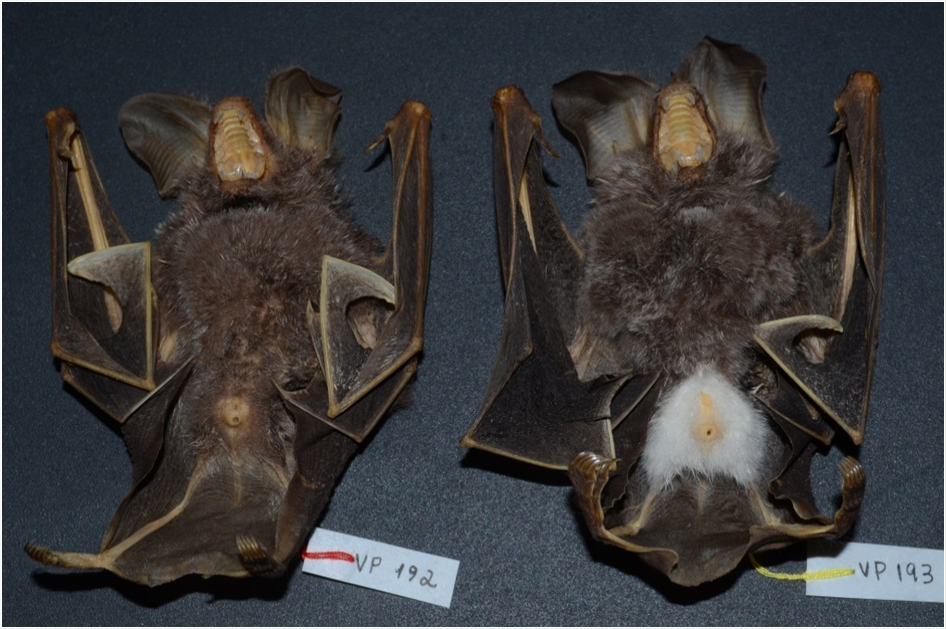
*Trachops cirrhosus* specimens: female (VP 192) and a piebald male (VP193)

A female and a male of *Trachops cirrhosus* were captured and deposited in the collection with the acronyms VP192 and VP193, respectively. The female specimen has a forearm measuring 59.9 mm and weighed 24 g, while the male specimen has a forearm of 62.4 mm and weighed 28 g. The male and female have a similar overall pattern, a characteristic grayish brown coloration. However, while the female shows no trace of chromatic disorder, the male has fur and skin of the ventral region, around the genital area, completely white (Fig. 1). Neither shows signs of reproductive activity.

## Discussion

Cases of pigmentation disorder reported in the literature are more frequent in closed habitats such as caves, mines, galleries, buildings and hollow trees (Uieda 2000). Such roosting strategies could enhancing their survival possibilities and increase the frequency of alleles for piebaldism. It also could be explained by the fact that both underground and urban roosts have been more intensively monitored and the number of aberrant bats in nature could be greatly underestimated (Kunz 1982). In this report, specimens of *Trachops cirrohosus* were captured when leaving their shelter in a cave, suggesting that such a roosting site could offer protection against predation. The species *T. cirrhosus* is a widely distributed and usually found during bat inventories (Gardner 2007). Due to this, the occurrence of aberrant coloration in this species seems to be very rare (Souza et al. 2013). The individuals of *T. cirrhosus* were captured in a highly fragmented habitat, and according to Bensch et al. 2004, individuals with piebaldism could be more common in small and isolated populations given that inbreeding increases the likelihood for recessive alleles to be expressed.

These phenotypes remain quite rare in wildlife. Nevertheless, this may have no effect on bats because they use echolocation and forage at night (Buys et al. 2002). As discussed by Souza et al. 2013, the occurrence of adult bats with this feature may mean that this property does not overly increase predation risk in the young to the point of never reaching adulthood. In fact, Brack and Johnson 1990 observed the same albino individual of *Myotis sodalis* for more than 5 years. Bartonička and Burič 2007 also observed the same albino individual of *Rhinolophus hipposideros* every year from 2000 to 2007. Sánchez‐Hernández et al. 2010 recaptured the same two albino specimens of *Desmodus rotundus* during 2 years and captured a lactating albino of the same species. Moreover, many authors reported the occurrence of pregnant or lactating bats with abnormal coloration (Brigham and James 1993 ; Talerico et al. 2008; Sánchez‐Hernández et al. 2010 ;García‐Morales et al. 2012; Lopez-Wilchis and Leon 2012; Rocha et al. 2013). These examples indicate that pigmentation disorders may not have a negative effect on reproductive sucess in these species.

## References

[B5289971] Abreu MSL., Machado R., Barbieri F., Freitas NS., Oliveira LR. (2013). Anomalous colour in Neotropical mammals: a review with new records for *Didelphis* sp. (Didelphidae, Didelphimorphia) and *Arctocephalus
australis* (Otariidae, Carnivora). Brazilian Journal of Biology.

[B5289983] Bartonička T., Burič Z. (2007). Records of the albino lesser horseshoe bats (*Rhinolophus
hipposideros*) in the Jeseniky Mts (Czech Republic).. Vespertilio.

[B5289993] Bensch Staffan, Hansson Bengt, Hasselquist Dennis, Nielsen Bo (2004). Partial albinism in a semi-isolated population of Great Reed Warblers. Hereditas.

[B5290003] Bilandžija Helena, Ma Li, Parkhurst Amy, Jeffery William R. (2013). A potential benefit of albinism in *Astyanax* cavefish: Downregulation of the *oca2* gene increases tyrosine and catecholamine levels as an alternative to melanin synthesis. PLoS One.

[B5290013] Brack V., Johnson S. A. (1990). Albino Indiana bat (*Myotis
sodalis*). Bat Research News.

[B5290033] Brigham R. M., James A. K. (1993). A true albino little brown bat, *Myotis
lucifugus*, from Saskatchewan.. Blue Jay.

[B5290043] Buys J., Heijligers H., Dorenbosch M. (2002). First record of an albino long-eared bat *Plecotus
auritus* in The Netherlands.. Lutra.

[B5290053] Caro TIM (2005). The adaptive significance of coloration in mammals. BioScience.

[B5290073] García‐Morales R., Tejera D. D., Ávila G. E. S., Moreno C. E., Akmentis M. S. (2012). Registro de leucismo en *Sturnira
ludovici* y *Artibeus
jamaicensis* (Phyllostomidae) en México. Chiroptera Neotropical.

[B5290127] Gardner A. L. (2007). Marsupials, xenarthrans, shrews, and bats. Mammals of South America.

[B5290141] Geiger D., Pacheco S. M. (2006). Registro de albinismo parcial em *Nyctinomops
laticaudatus* (e. Geoffroy, 1805) (Chiroptera: Molossidae) no sul do Brasil. Chiroptera Neotropical.

[B5290176] Guimarães M., Sato T., Kaku-Oliveira N., Uieda W. (2014). Primer registro de leucismo en *Artibeus
planirostris* (Spix, 1823) (Phyllostomidae).

[B5303676] Kunz Thomas H. (1982). Roosting Ecology of Bats. Ecology of Bats.

[B5290196] Lopez-Wilchis R., Leon G. M. A. (2012). A noteworthy case of leucism in *Artibeus
lituratus* (Chiroptera: Phyllostomidae) from Oaxaca, Mexico. Chiroptera Neotropical.

[B5290206] Lucati Federica, López-Baucells Adrià (2016). Chromatic disorders in bats: a review of pigmentation anomalies and the misuse of terms to describe them. Mammal Review.

[B5290216] Marín‐Vásquez A., Ortega‐Rincón M., Ramírez‐Chaves H. E. (2010). Records of leucism in three species of Colombian bats: *Carollia
brevicauda*, *Artibeus
jamaicensis* and *Lophostoma
silvicolum* (Phyllostomidae). Chiroptera Neotropical.

[B5290226] Rocha P. A., Feijó J. A., Donato C. R., Ferrari S. F. (2013). Leucism in Seba's short‐tailed bat, *Carollia
perspicillata* (Linnaeus, 1758), from a rock shelter in northeastern Brazil.. Chiroptera Neotropical.

[B5290236] Sánchez‐Hernández C., Romero‐Almaraz M. D. L., Taboada‐Salgado A., Almazán‐Catalán A., Schnell G. D., Sánchez‐Vázquez L. (2010). Five albino bats from Guerrero and Colima, Mexico. Chiroptera Neotropical.

[B5290248] Souza R. F., Novaes R. L. M., Felix S., Sauwen C., Jacob G., Santori R. T., Avilla L. S. (2013). First record of leucism in *Artibeus
lituratus* (Olfers, 1818) (Phyllostomidae) in Brazi. Chiroptera Neotropical.

[B5290261] Talerico Jennifer M., Jung Thomas S., Barclay Robert M R., Melton Kim S. (2008). Abberant coloration in a little brown bat (*Myotis
lucifugus*) from the Yukon. Northwestern Naturalist.

[B5290271] Treitler J. T., López‐Baucells A., Gomes Farias S., Tenaçol J. F., Rocha R. (2013). First record of a leucistic piebald *Phyllostomus
discolor* (Chiroptera: Phyllostomidae). Chiroptera Neotropical.

[B5290282] Uieda W. (2000). A review of complete albinism in the bats with five new cases from Brazil. Acta Chiropterologica.

[B5290292] Zalapa Silvia S., Guerrero Sergio, Romero-Almaraz María de Lourdes, Sánchez-Hernández Cornelio (2016). Coloración atípica en murciélagos: frecuencia y fenotipos en Norte y Centroamérica e islas del Caribe y nuevos casos para México y Costa Rica. Revista Mexicana de Biodiversidad.

